# A qualitative meta-synthesis of studies of patients' experience of exercise interventions in advanced cancer

**DOI:** 10.3389/fresc.2023.1298553

**Published:** 2024-01-04

**Authors:** Julie Young, Anna Lloyd, Erna Haraldsdottir

**Affiliations:** ^1^ St Columba’s Hospice Care Education and Research Centre, Edinburgh, United Kingdom; ^2^ Division of Nursing and Paramedic Science, Queen Margaret University, Edinburgh, United Kingdom

**Keywords:** exercise intervention, advanced cancer, experiences, palliative care, rehabilitation, meta-synthesis, qualitative analysis

## Abstract

**Background:**

People with advanced cancer often experience reduced functional capacity and quality of life. Research evaluating the potential benefit of exercise programmes for limiting such decline is accumulating. However, an appraisal of the evidence that considers the patient experience of exercise programmes, what mattered to them and what motivated and encouraged them to engage in exercise, has not been published. The aim of this meta-synthesis was to identify, appraise and bring together evidence from qualitative research in this area.

**Methods:**

Four databases were searched from 2nd January to 8th January 2023 for relevant studies. Qualitative studies investigating the experience of exercise as an intervention for adults with advanced cancer were included. Major findings and study characteristics were extracted. Findings were summarised, compared, and synthesised using meta-synthesis.

**Results:**

Eight studies were eligible and generated seven sub themes which informed the construction of three key themes: (1) *Impact of Delivery Method*; (2) *Emerging Motivation*; and (3) *Physical Impact*.

**Conclusion:**

The analysis revealed that exercise has the potential to positively influence all four dimensions of well-being: physical, psychological, social, and spiritual, for people with advanced cancer. Future research is required to consider the differential impact that the type, volume, and duration of exercise may have on the exercise experience for this patient group.

## Introduction

Dame Cicely Saunders advocated that people with advanced cancer should live as fully as possible until they die (1). A fundamental factor for achieving this goal is through retaining the capacity to stay as active as possible to maximise physical functioning. Substantial research has found exercise to be beneficial for the general cancer population (2). Exercise is key for optimising physical function and has also been shown to extend survival time after cancer diagnosis and treatment (3). More recently, there is an emphasis on the role of rehabilitation within palliative care, and growing evidence of the potential positive impact of exercise for people with advanced cancer (4). Benefits include increased Quality of Life, and reduced fatigue (5). In addition, exercise has been found to be safe and feasible within this population (5).

However, despite this evidence of benefits it is important to note that any intervention is embedded within the personal and social context of a person's life. Therefore, benefits may vary depending on this. Qualitative research methods can take account of such factors and provide comprehensive, detailed context-rich data on people with advanced cancer's perspectives of how they experience an exercise program.

Qualitative research exploring the experience of exercise in people with advanced cancer exists, however, bringing together what is currently known will provide deeper knowledge than would be gained from individual studies. Ultimately, this offers greater understanding of how, when or why any intervention may fit with a person's life circumstances (6).

## Methods

### PHASE ONE: selecting meta-synthesis and getting started

#### Aim of the meta-synthesis

The aim of this review is to systematically search, appraise and synthesise the qualitative research evidence of the experience of exercise as a structured intervention for people with advanced cancer. This will provide evidence of the perceived impact of exercise for this group of people, within the context of their personal and social life, that can inform future practice in palliative care. Exercise, for the purpose of this review is a structured exercise programme.

#### Rationale for using meta-ethnography

Meta-synthesis is a validated methodological approach for examining, critically comparing and synthesising qualitative research in a common topic (7–9). To guide the meta-synthesis, the study was informed by the seven step meta-synthesis process outlined by Noblit and Hare (10). Despite an increase in the use of meta-synthesis as a methodology, inconsistencies in the quality of reporting published meta-synthesis exist (11, 12). To maximise the impact of understanding the experience of exercise for people with advanced cancer, the eMERGE reporting guidance was used to support the presentation of the review teams seven step process (13).

### PHASE TWO—deciding what is relevant

#### Search strategy

In phase two discussions were held between members of the review team and the search strategy was defined. The Population, Exposure and Outcome (PEO) framework (14), supported the identification of key words which identified potentially eligible papers. Table 1 details the PEO format used for the search strategy.

**Table 1:** PEO format used to develop search terms (14).

Population		Exposure		Outcome
Palliative care OR End of life care OR Advanced cancer OR Metastatic OR Hospice	AND	Exercise OR Rehabilitation OR Physical therapy OR Exercise intervention	AND	Experiences OR Effects OR Outcome

#### Search process

To support transparency and the identification of relevant papers, the PRISMA checklist and four-phase flow diagram were used to guide the search process (15). The following databases were searched from 2nd January to 8th January 2023: CINAHL (Cumulative Index to Nursing and Allied Health Literature), MEDLINE (Medical Literature Analysis and Retrieval System Online), PsycINFO and PEDRO (Physiotherapy Evidence Database).

#### Selecting primary studies

Eligible papers met the following criteria: studies exploring adults with a diagnosis of advanced cancers experience of an exercise intervention; qualitative methods were used; written in English language. The following definition of exercise was used “Exercise is a subset of physical activity that is planned, structured, and repetitive and has as a final or an intermediate objective the improvement or maintenance of physical fitness” (16 pg. 126). The type and amount of intervention and supervision, intervention length or exercise dose did not influence eligibility. Papers were excluded if: participants were children (younger than 18 years old); participants were undergoing a pre-operative exercise programme or if they only had quantitative data; only explored health professionals/carers experiences; or were written in a language other than English.

Papers were screened through stages: (1) titles (2) abstracts (3) full texts. No restrictions were put on year of publication. Reference lists of all included studies were also screened. Abstracts from relevant conferences were hand-searched: ASCO (American Society of Clinical Oncology), ESMO (European Society for Medical Oncology), EAPC (European Association for Palliative Care), PRC (European Palliative Care Research Centre), ACSM (American College of Sports Medicine) and EMCC (European Multidisciplinary Cancer Conference).

**Figure 1:**
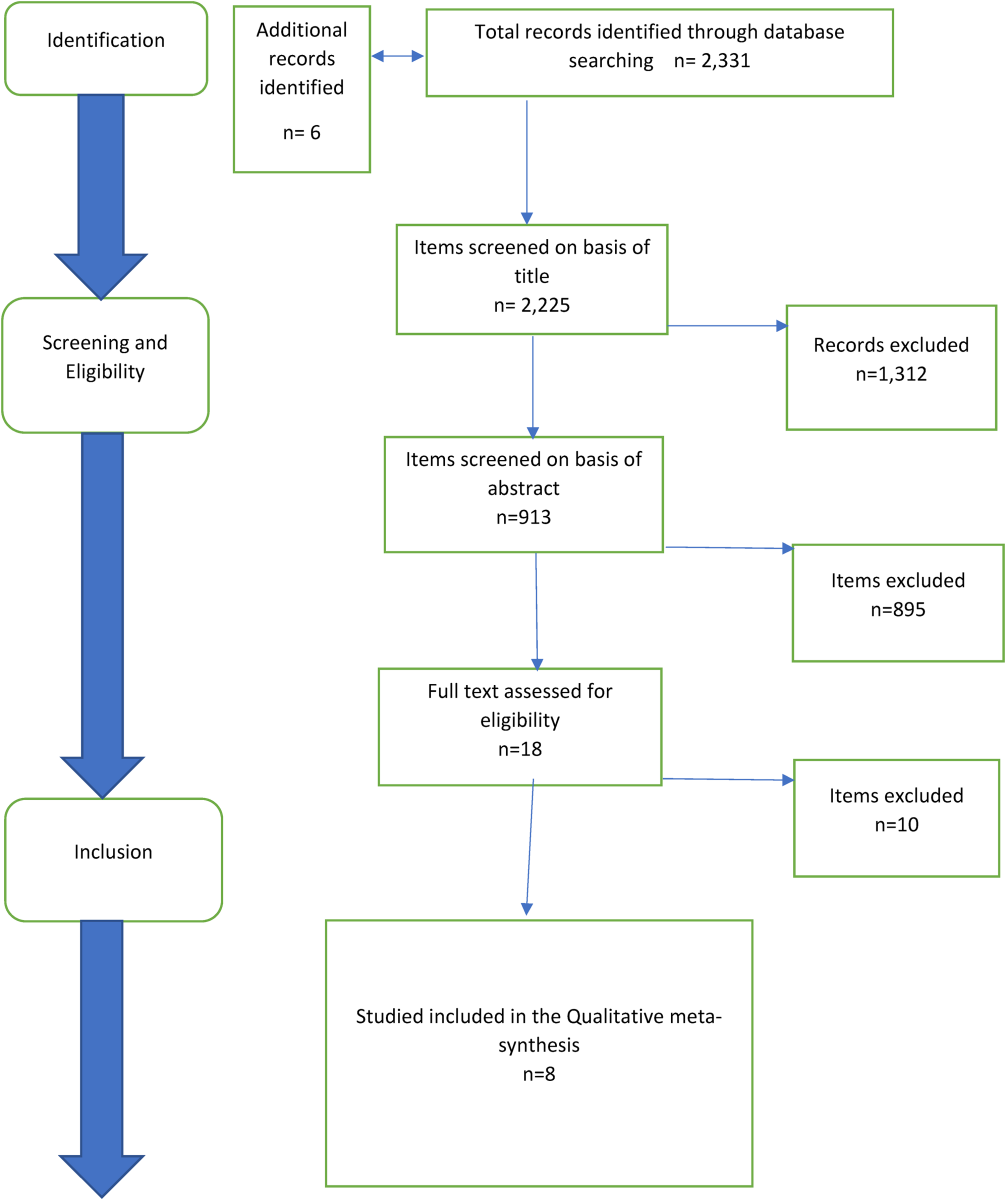
Flow chart of literature search process, identifying studies extracted, included and excluded at different phases of screening. Adapted from PRISMA (17).

The PRISMA flow diagram in Figure 1 visually summarises the screening process. The database search, conducted by one author (JY) identified 2,311 articles, and duplicates were removed. Following this, one author (JY) screened the titles and abstracts of the studies, and 18 full text publications were retrieved and screened. Upon reading the full text, a further 10 of these studies were excluded. 8 studies met the inclusion criteria.

### PHASE THREE—reading included studies

#### Reading and data extraction approach

The 8 included studies present qualitative data on adults with advanced cancers experience of an exercise intervention. During phase three, one author (JY) read and re-read the included papers, collecting data and identifying main findings from the primary studies. These were then shared with the two other members of the review team. Data concerning the characteristics of the study were extracted,—Table 2 summarises these.

**Table 2:** A summary of the papers selected.

Study & region	Aim	Sample size cancer site	Intervention	Setting	Qualitative methods	CASP score
Adamsen 2004 Denmark	To explore the nature of fatigue in cancer patients with advanced cancer undergoing chemotherapy on an exercise programme.	*n* = 23 Various sites (incl. Colon; Breast and Ovarian) Undergoing chemotherapy	6-week multi-dimension,4 × weekly group exercise programme Resistance and aerobic Also includes relaxation, massage, body-awareness, training	Hospital	Semi-structured interviews Thematic analysis	17
Adamsen 2012 Denmark	To explore the feasibility, health benefits and barriers of exercise in formerly sedentary lung cancer patients undergoing chemotherapy.	*n* = 15 Lung (87% NSCLC 13% SCLC) Undergoing chemotherapy	6-week, 2 × weekly, group exercise programme Resistance and aerobic Also includes a relaxation component	Hospital & Home	Semi-structured interviews and a focus group No identified method of analysis	18
Carr 2016 Cananda	To investigate the feasibility of home-based yoga to women with advanced cancer.	*n* = 3 All Female Various sites	3 sessions of 1:1 yoga with teacher Plus, independent use of a CD	Home	Semi-structured interviews Qualitative-content analysis	17
Midtgaard 2007 Denmark	To explore the experiences of advanced cancer patients participating in an exercise intervention and undergoing chemotherapy.	*n* = 5 Various sites (Inc. Breast; Ovary; Colon; Ewing sarcoma; non-Hodgkin's lymphoma)	6-week, group, 9hr/wk exercise programme Aerobic and resistance	Hospital	Unstructured diary analysis Phenomenol-ogical narrative	16
Mikkelsen 2022 Denmark	To explore patients’ experiences of participating in a multimodal exercise-based intervention in older patients with advanced cancer	*n* = 18 All 65 years or older Pancreatic or Biliary tract or non-small cell Lung Undergoing either chemotherapy or immunotherapy	12-week programme (1) Group exercise weekly (2) Protein drink before and after each session (3) Home-based walking programme (4) Nurse-led counselling Aerobic and resistance exercise	Hospital	Semi-structured interviews Evaluation questionnaire Thematic Analysis	19
Paltiel 2009 Norway	To understand the meaning and significance of a group exercise programme for advanced cancer patients.	*n* = 5 Colon or Rectal or Leimyosarcoma	6-week, 2 × weekly, group exercise programme	Hospital	Semi-structured interviews (Qualitative) and questionnaire (Quantitative) Phenomenol-ogical approach.	19
Payne 2018 UK	To explore patients’ and health professionals’ views and experience of palliative rehabilitation during advanced Lung cancer treatment	*n* = 8 Lung cancer Undergoing chemotherapy	6-week exercise and nutrition programme. Weekly telephone support. Aerobic and resistance	Home	Semi-structured interviews Thematic analysis	19
Turner 2016 UK	To explore patients’ experiences of an exercise programme within a palliative care setting.	*n* = 9 Various sites	Various lengths of time, individually tailored programme Aerobic and resistance exercise	Hospice	Semi-structured interviews Phenomeno-logical analysis	17

#### Presenting characteristics of studies

Studies were published between 2004 and 2022 and data was collected from 48 women and 37 men aged between 18 and 93 with varying types of cancers. Studies were conducted across Denmark, Canada, Norway and The United Kingdom. All studies collected data from people who had participated in a specific physical activity intervention. Interventions comprised of aerobic (*n* = 1); aerobic plus resistance (*n* = 6) and other exercise (*n* = 1). The intervention was delivered in various ways,—home-based; hospital or hospice based; Individual or group interventions.

#### Quality appraisal

One author (JY) assessed the quality of the studies included using the Critical Appraisal Skills Programme qualitative research checklist of questions (18). A score was assigned to each question (yes = 2; no = 0; and 1 = unclear), giving a maximum score of 20. Table 2 includes the overall scores for individual papers.. The decision was made not to exclude any studies from the meta-synthesis based on their quality or sample size. This is a relatively new and emerging area of research, where smaller scale studies could potentially offer insight to the review question.

Typically, those papers with a higher score provided greater detail around the process of data collection and analysis. Detailed accounts of ethical considerations were not always provided, impacting the integrity of those papers. The relationship between the researchers and participants was not always clearly reported, and there was an overall lack of detail on the authors' approach to issues of reflexivity during the data collection and analysis process, this raises issues over risk of bias. All studies provide a valuable contribution to understanding the experience of exercise for people with advanced cancer.

### PHASE FOUR—determining how the studies are related

**Figure 2:**
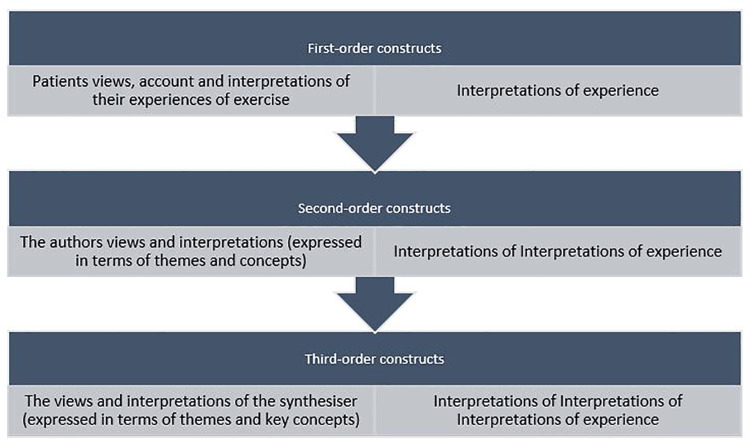
Definitions of first, second and third-order construct (10).

Relationships between the concepts originating from the studies were then examined by one author (JY) and then by the other two members of the review team. Instead of creating themes from raw data, the focus of meta-synthesis is to create what Noblit and Hare call third-order constructs (the key themes). These are drawn from second-order constructs (the authors themes) which are the themes identified by the studies’ authors, originally derived from first order constructs (the participants experiences) (10). Definitions are provided in Figure 2. The review team reached a consensus at each stage.

One author (JY) read and re-read the papers to note the second-order constructs. A table was compiled noting the second-order constructs in each paper, illustrated by raw data from the papers (first-order constructs). The table was then examined by the other two authors (AL and EH) and discussed as a review team.

### PHASE FIVE—translating the studies into one another

To begin the process of translating the terminology presented in the original studies, a list of all themes for all studies was created. Noblit and Hare highlight the role this plays in facilitating exploration; juxtaposition; and comparison (10). Translation describes the notion that author develops their own interpretative language to identify themes related to the review question. The creation of a new level of interpretation (third-order constructs), without further conceptual development (19). The meta-synthesis approach emphasises the contextual preservation of meaning within any given study (11). To support this, one author (JY) created a concept map, demonstrating the structure of the relationships between the concepts, and the recognition of how the second-order constructs related with one another. The map was examined by the other two authors (AL and EH) and discussed as a team.

Through this process, themes emerged (third-order constructs) and were used to summarise the information from the first and second constructs, while taking special care to preserve the original contextual meaning of the participants. Three key themes, and seven sub-themes, were identified.

## Results

### PHASE SIX—synthesising translations

Three key themes were identified from the eight studies. **Impact of the delivery method of the intervention** included sub-themes *programme setting, importance of social support* and *importance of professional support,* **Emerging motivation** included sub-themes *motivation to participate* and *new purpose,* and **Physical Impact** included sub-themes *improved physical function and health* and *managing the physical effects and symptoms of advanced cancer and its treatment.* Table 3 summarises the themes and sub-themes with quotations.

**Table 3:** Summary of the key themes, sub-themes and sample quotations identified in the studies.

Key-theme	Sub-theme	Relevant papers	Sample quotations
Impact of delivery method of intervention	Programme setting	(20–24)	“*[I]t wasn't an alternative to go back to the gym…in a gym where everyone's in good condition, that wasn't an alternative.”* (20)
	Importance of social support	(20, 21, 23–25)	“*Mentally, I wasn't in the best condition, so I preferred to get together with others with the same sort of thing*.” (20)
	Importance of professional support	(20, 23, 24, 26)	“*So, there is an expectation that you will do your best to get whatever is able to be got out of you, that you are going to do it and…whatever you achieve is an achievement and that is a psychological boost*.” (23)
Emerging motivation	Motivation to participate.	(20, 21, 24, 26, 27)	“*Very reassuring, you know what I mean. I knew I could have lifted the phone if I wasn't happy, and you’d be there. Thank God I didn't have to do that. But I looked forward [the weekly phone call]…it did [motivate me].”* (26)
	New purpose	(20, 22, 23, 25, 26)	“*I reached my goal today. I now know that I can do things, eg. Jog or something similar when the project finishes*.” (25)
Physical impact	Improved physical function and Health.	(20–27)	“*Now I have normal strength in my legs, and it is confirmed that I didn't when I began the program*.” (21)
	Managing the physical effects and symptoms of advanced cancer and its treatment	(21–24, 27)	“*In terms of physical this last chemo, the improvement that I have felt, the yoga helps me to rebound.”* (22)

Each key theme captured a different dimension of the experience of exercise and together suggest a positive impact on participants not only physically but also socially, psychologically, and spiritually.

### PHASE SEVEN—expressing the synthesis

#### Impact of the delivery method

This theme captures the influence, on participant experience, of the specific method chosen to prescribe exercise, specifically, where how and by whom it is delivered.

##### Programme setting

The setting for the exercise was relevant, with five studies highlighting the importance of the location of the intervention for patients (20–24). Four of these identify the hospital/hospice setting as instrumental in the participants' experience of the intervention (20, 21, 23, 24) with all 15 participants in Adamsen et al.'s study reporting they lacked the initial motivation to exercise at home (21).

Participants found the hospital environment to be practical as visits could be combined with other health care appointments and provide access to physiotherapists from whom feedback was appreciated (20, 24). In addition, they were reassured by being in an environment where there was an understanding of illness and fluctuating performance from day to day. Mikkelsen et al. reported “some” of the 18 participants in their study appreciated the opportunity to co-ordinate visits for exercise programmes with medical appointments, positively influencingtheir capacity to take part because, even on their “bad days”, they had to attend hospital appointments (24). In Adamsen et al.'s study of a home and hospital-based exercise intervention participants reported that although the program was easily comprehended, several reasons existed for why they struggled to follow it (21). Shortness of breath, severe fatigue and a general “bad mood” as well as the absence of their exercise partners acted as substantial barriers to the self-discipline, they felt they needed. Furthermore, participants were dubious that any benefits from home-based exercise would equate to those likely with supervised sessions.

Conversely, all participants in Carr et al.'s home-based study found value in exercising in their own home (22). Participants outlined the benefit of a home-based intervention describing the advantages of this method of delivery for enhancing their time at home (22). One participant described home-based delivery making such a difference yet no reason for this was explored within the paper. However, this study involved only three participants and was atypical in providing one-to-one home exercise sessions with an instructor, which almost certainly impacted on the experience.

##### Importance of social support

Exercise facilitated social connections (20, 21, 23–25), by providing opportunities for new interactions. The group environment was reported as helping to reduce feelings of *social isolation* (20, 21, 23) and increase feelings of *relatedness* (20, 21) *belongingness* (20, 23) and *camaraderie* (23). Adamsen et al. highlight the importance of the group setting for the delivery of an exercise intervention (21), and Midtgaard et al. explain how participants developed an accessible and meaningful community with a shared feeling of *self-reliance* (25). However, neither paper explored whether these positive changes were, or could be, maintained post-trial.

Paltiel et al. described how the commitment of group participation was motivational. An informal support system developed between participants, however, given that the relationships developed were non-committal, this allowed the option of anonymity to be retained if desired (20).

Two participants in Turner et al.'s study expressed sadness when discussing the decline in health or death of peers in the group, however, most remained encouraged and supported by these relationships (23). This study did not record the length of the intervention. This may be an important implication given the greater likelihood of other participants dying during the intervention in longer trials.

Some participants in Mikkelsen et al.'s study described feeling that initial interactions with others in the group felt slightly confrontational yet, over time, this dissipated with accounts relating to the growing importance of sharing experiences and mutual support (24).

##### Importance of professional support

The support of the [teacher] was identified as important in four studies (20, 23, 24, 26). Paltiel et al.'s study reported that all participants felt the exercise group should be led by a health professional with knowledge and training in physical activity in addition to a knowledge of cancer (20).

Participants' experience of exercise was also influenced by the engagement of professional staff that they described as something that contributed to a *sense of belonging* (23). Professionals' attitudes of *encouragement without expectations* could be pivotal in maintaining positivity during exercise. In addition, a patient's decision to participate was heavily influenced by their understanding that exercise would be individually tailored (20). Mikkelsen et al.'s study offered nurse-led support and counselling based on individual need (24), this was appreciated by participants. Some of the oldest and frailest participants found value in this.

#### Emerging motivation

Underpinned by two sub-themes (Motivation to Participate and New Purpose) this theme captured what motivated those with advanced cancer to participate in exercise, how this can expand or shift during the experience and can lead to developing a new sense of purpose.

##### Motivation to participate

Four studies identified motivation to participate in exercise as a theme (21, 24, 26, 27). Factors that motivate people to participate in exercise as well as factors that demotivate people to participate in exercise were identified.

A desire to reduce the physical burden of chemotherapy and illness was identified as a motivating factor. Seventeen of the 23 participants in Adamsen et al.'s study hoped exercise would reduce their growing levels of fatigue (27) while those in Adamsen et al.'s study where motivated by the potential to increase their physical fitness but also hoped that participation in the programme might improve their life expectancy (21). Participants described how motivating it was to have new, attainable goals whilst their ability to exercise fluctuated.

Group membership became a primary motivational factor that encouraged the participants to reach a goal or make progress beyond their physical limitations (20, 21). As such, the person's interest in continuing to participate in the group affirmed their desire to improve. In Mikkelsen et al.'s study, participants highlighted the value of laughing and enjoying themselves in motivating them to continue to exercise (24).

The opportunity to exercise in a secure environment under the supervision of health professionals was a motivating factor. After commencing the exercise intervention, three of the five participants in Paltiel et al.'s stopped attending their public gym and chose not to return (20). They described feeling that their physical appearance was incompatible with the traditional gym environment and that the knowledge and understanding of the gym personnel regarding their condition was inadequate to be able to support their specific needs.

In Payne et al.'s study, the exercise was solely home-based, and participants were given weekly telephone calls from a health professional (26). They described how this framework encouraged them to continue to exercise. What may also be salient is that accountability to health care professionals was perhaps a motivating factor. Conversely, in Adamsen et al.'s study where the intervention was a combination of hospital-based supervised group intervention and an unsupervised, home-based component, participants were less engaged with exercise at home. All 8 participants identified a preference for hospital based, supervised, structured group training (21).

Two of the four studies that identified motivation to participate as a theme reported that motivation to participate expanded over the duration of the intervention. When participants did not continue with exercise, they attributed it to their insecurities about the appropriate level of exercise for their diagnosis and treatment (27).

##### New purpose

Exercise was able to offer a sense of “orientation” through providing purpose and coherence in everyday life at a time when this may have been lost due to the illness (20, 22, 23, 25, 26). For example, in Turner et al.'s study, participants could recognise the value of taking part in an exercise intervention which enabled them to maintain as functional a capacity as possible for as long as possible (23). Some of their 9 participants described how the exercise gave their lives a new value that encouraged them to take a more active role in their everyday lives and increased feelings of autonomy (23). These factors were important in fostering hope when participants' diagnosis resulted in a feeling of loss of control over their own body and health (23, 25).

Exercise cultivated a variety of positive self-perceptions. Individuals in Turner et al.'s study described themselves using words including proud, accomplished and confident following exercise sessions (23). Some participants in Paltiel et al.'s study described how having the opportunity to talk to people in similar circumstances supported them to stretch themselves to their own limits (20). Furthermore, participants in Paltiel et al.'s study reported that exercise related to increased well-being and provided distraction from depressive thoughts. Other studies described how positive self-evaluations extended to life beyond the intervention with several studies highlighting exercise as helping to reduce negative feelings and thoughts (20, 22, 23, 25). Additionally, Carr et al.'s study reported that participants described positive emotional responses, however, caution should be noted as this latter study involved only three participants receiving home-based yoga sessions (22).

Finally, following the exercise intervention participants reported feeling encouraged to interpret their situation as one that demanded *self-action*, engendering an increased sense of personal agency (20, 23, 25, 26).

#### Physical impact

Underpinned by two sub-themes of improved functional and physical health and managing the physical effects and symptoms of the disease and its treatment this theme captured participants' perceptions of their physical gains from participating in exercise as an intervention.

##### Improved functional and physical health

In seven of the eight studies, individuals reflected on the physical benefits they experienced through exercise, noticing improvements in their health and physical functioning. Exercise is reported as helping participants to feel physically better (increased strength and vitality) inhibiting perceived health anxieties and improving everyday functioning. Authors outlined specific benefits to participants in relation to physical fitness (20, 21, 23, 24, 26, 27); physical strength (21, 23, 24, 27); overall energy levels (21, 22, 24, 27); functional mobility (23); flexibility (22); pain (21, 22) and overall physical well-being (21, 24). Adamsen et al.'s study reported that 92% of participants described improved physical well-being and surplus energy in the day following exercise (27). Midtgaard et al. described how taking part in the exercise intervention led some participants to challenge their understanding of what their physical capacities and limitations were (25). Participants described how they confronted and challenged their limitations through experiencing improvements in physical strength and expending energy in a “purposeful manner”. Doing so encouraged feelings of physical agency where participants felt increasingly able to do things they want to do.

Some participants, however, describe negative experiences relating to physical discomfort (21, 25). Six participants in Adamsen et al.'s study reported physical discomfort, however authors attributed this to not having undertaken any exercise at all prior to the study (21). In Midtgaard et al.'s study, one participant reported uncertainty surrounding pre-existing physical discomfort and their capacity to exert themselves (25).

##### Managing the physical effects and symptoms of advanced cancer and its treatment

Participants outlined how exercise assisted them to manage the physical ramifications of their disease, which, in turn, enhanced their physical quality of life and ultimately impacted positively on their emotional well-being.

Studies reported that exercise has the potential to alleviate, in some part, the symptoms of cancer and its treatment. Specifically, exercise enabled participants to take a degree of control of their cancer related fatigue (21, 23, 27) and pain (21, 22). Participants in Carr et al.'s study described how exercise relieved chemotherapy related symptoms of *reduced flexibility, nausea and fatigue* (22)*.* However, in Mikellsen et al.'s study, fatigue was identified as a barrier to exercise, with some participants highlighting it as the main reason for non-adherence (24).

Participants in Adamsen et al.'s study discussed their experiences of exercise induced a different sense of fatigue that was more like tiredness that was familiar from pre illness life (27). This feeling was associated with life enhancing activities and contrasted starkly with their experience of fatigue that was a consequence of cancer which held negative associations of illness, physical pain, existential concerns, and no association with potential benefit. Adamsen et al.'s study focused specifically on the nature of fatigue through exercise. Participants described this new experience of fatigue, describing it as exercise-induced fatigue, which, to some degree, counter-balanced the negative effects of disease/treatment induced fatigue (27). However, it is important to note this was one of only two studies offering participants massage, relaxation, relaxation, and body-awareness training in addition to aerobic and resistance exercise training. It remains unclear what impact these additional interventions had on the participants' experience.

In contrast to the more positive evaluations above, in Mikellsen et al.'s study participants described having sore muscles after exercise and two acquired exercise related injuries (24). Although many positive physical responses to exercise programmes are described, only five of these studies reported perceived physical benefits of exercise as an outstanding theme with the remainder highlighting non-physical outcomes (21–24, 27).

## Discussion

At present the palliative regime only enables a small percentage of people with advanced cancer to exercise (28). With the recent emphasis on rehabilitation in palliative care (29, 30) promoting exercise in clinical practice may well encourage people with advanced cancer to focus on optimising well-being amidst ill-health.

This meta-synthesis outlines the value of exercise for people with a diagnosis of advanced cancer and provides an understanding of its impact at a personal level. The eight studies reviewed support the premise that exercise can yield a range of perceived benefits with a positive impact on well-being extending beyond the purely physical. This allies with the holistic approach of palliative care with the fundamental belief that *physical, social, spiritual*, and *psychological* needs are intertwined (31). Consequently, whilst exercise might be seen as having a positive impact on physical wellbeing, the evidence from this review highlights the integrated impact of exercise.

Findings of this review suggest that people with advanced cancer who participate in exercise have the potential to establish meaningful personal reward from perceived improvements in strength and functional status, such as, an ability to climb stairs with greater ease. Improvements in the functional status of this group have been shown to correlate with more integrated impact as increases in quality of life (32).

Our findings also suggest that exercise can help reframe the experience of fatigue for people with advanced cancers, from an “emotionally negative disease related symptom” to a more “emotionally positive response” to exercise. These findings correlate with the Body and Cancer study, where a reduction in fatigue was affected by an increase in vitality (33). When diagnosed with advanced cancer, the associated distress and uncertainty can activate a search for new meaning in life (34, 35). Thus, the capacity of exercise to enable and encourage a *sense of purpose* when faced with an intractable illness can be a helpful factor in coping in the aftermath of such a diagnosis. Further, when participants perceived themselves as achieving their goals, such as maintaining a higher level of function, they expressed feelings of increased hope. Hope is a factor that is important in enabling people “adjust to illness, reduce distress, and improve quality of life” (36 p. 67) and is important in maintaining the well-being of people living with advanced cancer (37, 38). However, establishing and maintaining hope when faced with a diagnosis of cancer is arduous when facing treatment side effects, hospitalisation and a loss of control (39, 40). By participating in structured exercise as an intervention, participants were offered new possibilities and *hope*, including the drive to obtain *positive outcomes*.

People with advanced cancer can feel forced to submit to a specific set of circumstances, for example, having no option for seeking a cure as well as increasing dependence on others as the illness progresses (41). Such circumstances can potentially diminish autonomy (42), fostering despair and a loss of feelings of being in control. Exercise can restore a perceived sense of predictability and “sense of control” in one's life, a factor identified as crucial to positive self-rated health in the face of illness (43).

Taking part in exercise offers people with advanced cancer the opportunity to consider themselves as undertaking what is traditionally regarded as a health promoting activity with the positive associations that the subsequent fatigue can bring. That people can experience these positive gains from a relatively normalised activity resonates with the theory of Health-within-illness where the states of health and illness are not mutually exclusive and that one may experience feeling “healthy” even while living with advanced cancer (44). This benefit to overall wellbeing should not be underestimated.

Motivation is a critical factor in reinforcing and sustaining exercise (45). The positive influence the group setting has on motivation was emphasised in most studies; however, being part of a group could provide social support. It should be noted that, given that most programmes were time-limited, and hospital/hospice based, the loss of the group as a motivating factor could explain the low levels of post intervention adherence. Accordingly, finding ways for people to continue group-based exercise and thus maintain both motivation and social contact could allow the maintenance of the perceived benefits beyond the time frame of an intervention.

Despite describing holding negative views of hospitals (46) and hospices (47), participants appreciated the opportunity to exercise in these environments as they included the presence of a health professional, where physical limitations and treatments are understood, handled sympathetically and without judgement and where those involved understand the mental and physical toll of advanced cancer. This is an important point because treatment-related symptoms (48) as well as symptoms of the disease (49) have been found to prevent people from participating in exercise due to anxiety that exercise may cause harm. Only a small number of studies delivered exercise in the home environment limiting the capacity for adequate comparison.

People are living with advanced illnesses, including cancer, for much longer than previously (50). This review suggests that exercise offers an adjunct to palliative care through the potential to reduce disease symptoms, foster hope, provide opportunities to connect with others, offer a sense of purpose and improve well-being. Exercise may therefore reduce the overall burden of living with advanced cancer. Furthermore, it offers the opportunity to improve, maintain or lessen the loss of physical strength and thus protect against losses to functional dependence as illness progresses. This is a highly salient factor for the well-being of those living with, and dying from, advanced cancer.

## Limitations of findings

Throughout the studies, there was little detail provided on individual stages of the participants' advanced cancers. Where metastases were recorded, site was not specified, excluding the opportunity to explore the influence of this on participants ability to participate in the interventions. In addition, there was little detail on the *type of exercises* and how it may impact the value of the exercise to patients. Similarly, while there was a clear preference for a tailored exercise programme, little was recorded about dosage (*frequency, intensity, and duration*) of the intervention which may impact on its value for participants.

The structure of the programmes could impact on the feasibility and sustainability of the exercise intervention, and whether the outcomes highlighted in this review can be maintained in the long-term is not clear.

Meta-synthesis research has been criticised for its reductive tendencies (51, 52). Focusing on common themes may have reduced the complexity minimising the important context of the original study findings thus minimising differences between them. Whilst the results from this review were overwhelmingly positive there were some negative experiences reported: guilt was experienced by some when not achieving goals; some lacked the discipline to exercise at home; some reported sadness when seeing others in the group deteriorate or die; and some experienced physical discomfort and injury.

It should be acknowledged there is likely to be a bias inherent in the studies towards positive evaluation due to participant selection. Samples may not have been representative of the intervention group, some studies reported patients dying between the end of the intervention and commencement of the interviews.

There may have been individuals who participated in the exercise intervention but declined to be interviewed for qualitative research, perhaps due to dropping out of the programme or because they had more negative experiences. In the studies that reported on attrition, the majority who withdrew from the exercise interventions were mostly due to disease progression and it was not deemed ethical for those participants to continue to the qualitative interviews.

Similarly, it is likely that studies only recruited people who were initially motivated enough to exercise and thus cannot account for the views of those who declined to participate. In addition, most participants were still being treated for their advanced disease and therefore had a degree of functional capacity. Finally, some studies had insufficient participant numbers and inadequate data, reducing the scope and transferability of the findings.

## Implications for research

In recent decades, exploration of people with advanced cancer's perspectives on the impact of exercise in both qualitative and quantitative studies, has increased. However, this synthesis highlights gaps in the research literature.

First, little literature exploring differences in types of exercise exists. In general, exercise is recognised as a complicated behaviour comprising leisure time, occupational, commuting and household activities (53). Further research is required to explore the differential impact of these various types of exercise for people with advanced cancer.

Second, further research is needed to develop an understanding of the experience of people with advanced cancers regarding the frequency, intensity, and duration of an exercise intervention. This is likely to influence the value of the exercise and thus its impact, as well as participation in and attrition during a specific intervention study.

Thirdly, comparison of individual vs. group-based exercise would be beneficial. Further research should consider the risk of developing a degree of dependency on the support from health professionals and peers for those in group-based programmes as this would have important implications on the sustainability of the intervention.

In all studies, participants were instructed to comply with a specific intervention. The provision of greater detail about the specific interventions delivered within future research would be beneficial. However, allowing individuals to determine the frequency, duration, and intensity of their exercise is a developing approach which has the potential to lead to psychological and emotional improvements (54). Consequently, further research could establish if this may be applicable to people with advanced cancer. Finally, studies with protracted follow-up evaluations could determine if any value is sustained over time.

## Conclusion

This study offers a synthesis of qualitative literature regarding the experience of taking part in structured exercise interventions for those with advanced cancer. The findings highlight that those that took part in the studies can perceive benefits of exercise that extend beyond physical elements. They also describe what is important to people with advanced cancer when engaging in physical exercise and how this information can be used to tailor exercises to the needs and wishes of the people to reach the optimal result. Exercise interventions, especially in a group setting, can contribute to new physical and emotional states where cancer can co-exist with activities and behaviours associated with promoting health.

These findings offer information about the potential components that influence initial, as well as continuing participation in exercise programmes. Thus, group exercise programmes delivered to people with advanced cancer by suitably trained health professionals could be developed within the hospital/hospice environment as part of routine palliative care, reducing symptom burden, increasing physical, social, emotional, and spiritual well-being.
